# Latest Advancements in the Management of H3K27M-Mutant Diffuse Intrinsic Pontine Glioma: A Narrative Review

**DOI:** 10.3390/cancers17030420

**Published:** 2025-01-27

**Authors:** Maria Chiara Lo Greco, Giorgia Marano, Madalina La Rocca, Grazia Acquaviva, Roberto Milazzotto, Rocco Luca Emanuele Liardo, Antonio Basile, Pietro Valerio Foti, Stefano Palmucci, Emanuele David, Silvana Parisi, Antonio Pontoriero, Stefano Pergolizzi, Corrado Spatola

**Affiliations:** 1 Radiation Oncology Unit, Department of Biomedical, Dental and Morphological and Functional Imaging Sciences, University of Messina, 98122 Messina, Italy; giorgiamarano@gmail.com (G.M.); madalina.larocca@gmail.com (M.L.R.); silvana.parisi@unime.it (S.P.); apontoriero@unime.it (A.P.); stefano.pergolizzi@unime.it (S.P.); 2 Radiation Oncology Unit, University Hospital Policlinico “G. Rodolico-San Marco”, 95123 Catania, Italy; gra.acquaviva@libero.it (G.A.); r.milazzotto@policlinico.unict.it (R.M.); lucaliardo@hotmail.com (R.L.E.L.); cor_spatola@hotmail.com (C.S.); 3 Department of Medical Surgical Sciences and Advanced Technologies “G.F. Ingrassia”, University of Catania, 95123 Catania, Italy; basile.antonello73@gmail.com (A.B.); pietrofoti@hotmail.com (P.V.F.); spalmucci@unict.it (S.P.); david.emanuele@yahoo.it (E.D.); 4 Radiology I Unit, University Hospital Policlinico “G. Rodolico-San Marco”, 95123 Catania, Italy

**Keywords:** diffuse intrinsic pontine glioma, midline glioma, central nervous system, radiotherapy, chemotherapy, target therapy, immunotherapy

## Abstract

Despite recent advancements in radiotherapy for Diffuse Intrinsic Pontine Glioma (DIPG), the prognosis of this disease remains poor, highlighting the need for new treatment strategies to improve outcomes. Adding stereotactic biopsy to the diagnostic process for children with DIPG has been crucial in improving the management of this disease. Indeed, the discovery of the H3K27M mutation as a key driver of DIPG has led to the development of new drugs that are more effective than traditional ones. These include nimotuzumab (an anti-EGFR drug) and vinorelbine (a semisynthetic vinca alkaloid) in combination, Panobinostat (a histone deacetylase inhibitor), ONC201 (a drug that blocks the dopamine receptor D2 and inactivates Akt and ERK kinases), and chimeric antigen receptor (CAR) T cells. In terms of local therapy, identifying the H3K27M mutation can help us explore how genetic changes affect treatment response, recurrence patterns, and survival. Beyond the time to first recurrence, specific patterns of tumor recurrence, like leptomeningeal spread, can influence treatment plans. For example, radiotherapy can be adjusted in terms of doses and volumes, based on tumor aggressiveness. Because the H3K27M mutation is linked to higher malignancy, a slightly higher dose could be used for the second round of local irradiation. Additionally, irradiating the entire craniospinal axis could help control both local and leptomeningeal disease.

## Simple Summary

The introduction of stereotactic biopsy in the diagnostic pathway of Diffuse Intrinsic Pontine Glioma (DIPG) has been pivotal in enhancing the understanding of this aggressive malignancy and fostering the development of innovative treatment strategies. Various systemic therapies, including nimotuzumab and vinorelbine, Panobinostat, ONC201, and CAR T-cell therapies, are currently under investigation. Radiotherapy remains the cornerstone for both primary and recurrent DIPG, offering the potential to tailor treatment based on tumor characteristics, such as doses and volumes, thereby optimizing therapeutic outcomes. This narrative review aims to present an overview of the latest advancements in DIPG management to support a better understanding and further research aimed at improving survival rates for this disease.

## 1. Introduction

Diffuse Intrinsic Pontine Glioma (DIPG) is the most prevalent brainstem tumor of childhood, representing approximately 75–80% of all brainstem neoplasms in this age group [1].

Given its peculiar localization in such a critical site as the brainstem, surgery is not generally feasible, and the only local treatment at our disposal, both for newly diagnosed and recurrent DIPG, is radiotherapy [2]. As reported in one of our previous experiences, more than one course of radiotherapy, although not curative, represents a feasible and safe approach, able to improve overall survival (OS), disease-free survival (DFS), and quality of life (QoL) in selected patients [3].

However, despite advancements in local therapy, DIPG remains a fatal disease, highlighting the need to discover new therapeutic targets that can overcome radioresistance and enhance treatment efficacy. In this context, the increasing importance of molecular diagnostics in DIPG has been pivotal in advancing the management of this malignancy, with many novel therapeutic strategies currently under investigation.

The aim of this narrative review is to present an overview of the latest advancements in DIPG management. This includes the integration of stereotactic biopsy into the diagnostic protocol and the subsequent discovery of the H3K27M mutation, with a special focus on its therapeutic and prognostic impact on the disease.

## 2. Advanced Diagnostic Strategies for DIPG

Traditionally, DIPG diagnosis has mainly been based on clinical features and neuroimaging findings. In fact, at first observation, children usually present neurological symptoms such as ataxia, pyramidal tract dysfunction, and an abducens nerve (cranial nerve VI) palsy, suggestive of brainstem involvement [4].

Neuroimaging with contrast-enhanced MRI is generally performed afterward, to confirm the presence of an intrinsic, pontine-based infiltrative lesion, possibly encasing the basilar artery [5]. With MRI, the tumor’s aspect is characterized by hyperintensity at T2-weighted and FLAIR sequences and hypointensity on T1-weighted sequences; however, areas of enhancement could be visible, especially around foci of necrosis. With regard to advanced MRI sequences, a literature report demonstrated that apparent diffuse coefficient (ADC) images may help identify prognostic groups, with improved survival being associated with high ADC values (>1300) [6].

Despite the consistency of imaging techniques in DIPG diagnosis, recent experiences have suggested that MRI is sufficiently reliable only in the cases of typical DIPG, whereas atypical presentations may need histological confirmation.

Indeed, analysis of MRI consistency revealed a significant amount of variation among pediatric neurosurgeons, especially in atypical tumor presentations, leading to a lack of standardization in DIPG diagnosis and treatment [7].

With the modernization of surgical techniques (i.e., stereotactic technology and operative microscopes) and the growing role of molecular diagnostics in DIPG, the relevance and safety of brainstem biopsies have been highlighted. This step helped move the field forward, leading to the incorporation of stereotactic biopsy in all children with suspected DIPGs in the diagnostic protocols [7].

Indeed, even if biopsy was historically considered unnecessary to establish a DIPG diagnosis, nowadays, studies show that this procedure can be performed safely and could be used to enhance diagnosis and support research requiring histological and molecular data [8].

Besides information on molecular phenotypes derived from the acquisition of malignant tissue through tumor biopsy, further driver mutations are detected in circulating DNA (ctDNA) from peripheral blood, enabling reliable monitoring during and after treatment with a decreased content of ctDNA if the tumor has receded. Therefore, liquid biopsies could be potentially used as an alternative to biopsy to analyze these biomarkers in blood and cerebrospinal fluid (CSF) [9,10,11].

## 3. Genomic Profiling

Most midline high-grade gliomas carry the H3K27M mutation and are therefore identified, according to the WHO 2021 classification, as diffuse midline gliomas, “H3K27M mutant” [12]. This mutation is not only a molecular marker but also confers a WHO Grade 4 designation, regardless of histological features, reflecting its aggressive nature. This recent classification encompasses most of the DIPGs, as over 80% of them are characterized by this mutation [13].

The H3K27M-mutant subgroup is characterized by a missense mutation in histone 3, specifically in position 27 where lysine (K) is replaced by methionine (M). In this context, H3F3A (H3.3) and HIST1H3B/C (H3.1) are two known mutation variants and play a significant role in the overall functional characteristics of DNA [14].

In detail, H3K27M mutation is correlated with a global epigenetic alteration, traditionally thought to result from the deactivation of the polycomb repressive complex 2 (PRC2), via the zest homolog-2 (EZH2) enhancer and the mutant histone interaction [15]. However, recent research has demonstrated the presence of heterotypic nucleosomes and retained PRC2 activity, suggesting that the mutation does not fully abolish PRC2 function but instead modulates its activity in a more complex manner [16].

From the clinical point of view, patients with H3 mutations tend to exhibit a more aggressive clinical course and a worse response to radiation therapy. Although DIPG itself is already known to be fatal due to its location, further studies have shown that H3K27M mutants have an even smaller OS, with worse outcomes in the H3.3-mutant population compared to H3.1. Specifically, in 2017, Mackay and colleagues conducted a meta-analysis showing that OS for H3K27M-mutant DIPG was 2.5 months shorter compared to the wild-type subgroup [17,18,19].

Beyond H3K27M, other concomitant genetic alterations have been detected, influencing the regulation of embryonic morphogenesis, the activity of transcription factors, and cellular growth. Among these, we report alterations in TP53, ACVR1, components of the PI3K/mTOR/MAPK pathways and ATRX [20].

The second-most common mutation in H3K27-mutant DIPGs is TP53 mutation, which occurs in about 40% of DIPGs and relates to a worse OS [21,22]. This mutation consists of the loss of normal p53 protein function, which impairs cells’ ability to undergo apoptosis in response to DNA damage. As a result, cells with damaged DNA can continue to proliferate, contributing to tumor growth. Additionally, TP53 mutations are associated with increased resistance to radiotherapy, making treatment more challenging [23].

Furthermore, it has been shown that H3K27M-mutant and TP53-mutant DIPGs are characterized by superior radioresistance, increased tumor aggressiveness, and consequently worse OS, compared to the subgroups without the mutations or with only one out of the two [24,25].

Activin A receptor type 1 (ACVR1) is a component of the bone morphogenic protein signaling pathway and has been identified in nearly one-third of DIPGs. This gene encodes a receptor involved in the bone morphogenetic protein (BMP) signaling pathway, which is important for cell growth and differentiation [26]. In normal conditions, ACVR1 helps with myelination within the central nervous system. However, when mutated, it encodes a serine/threonine kinase (ALK2) receptor which has an enhanced sensitivity to the ligand activin A, and therefore impacts the dysregulation of the BMP/SMAD pathway, increasing tumor proliferation. ACVR1 mutation has been found to be associated with an earlier tumor development onset, with a median age of 5 years at diagnosis, and a slightly longer OS (15 months) [25,26,27].

The mutation of PIK3CA is present in around 15–20% of DIPG cases. As this gene encodes a subunit of the PI3K enzyme, which is involved in cell growth, proliferation, and survival, its mutation results in the activation of the PI3K/AKT/mTOR pathway, supporting tumor growth and survival [28].

ATRX genetic mutations are present in around 9% of DIPG and are characterized by a high co-occurrence rate with the H3K27M mutation. In this context, the loss of the complex appears to be associated with a destabilization of telomeres and altered gene expression, which in conjunction with the H3K27M mutation contributes to tumor development [19,29].

Among H3K27M mutants, other co-occurrent alterations have been found, including TP53, platelet-derived growth factor receptor (PDGFRA) amplification, and ACVR1 genetic alterations. However, the clinical meaning of this event is still unknown, and further research is needed to better interpret the heterogenous genetic landscape of H3K27M-mutant tumors [29].

## 4. Novel Systemic Agents for H3K27-Mutant DIPG

The administration of chemotherapy agents in patients with this malignancy has historically represented a major challenge for clinicians, due to the high expression of O6-methylguanine DNA methyltransferase (MGMT) by DIPG, which makes it particularly chemoresistant to temozolomide [30,31].

Fortunately, the discovery of the hallmark H3K27M alteration as the major driver of DIPG has inspired the development of novel agents that have demonstrated improved efficacy compared to traditional drugs.

The combination of nimotuzumab (anti-EGFR) and vinorelbine (semisynthetic vinca alkaloid) for DIPG already showed encouraging results in terms of median progression-free survival (PFS) and OS in a pilot phase II study published in 2014 [4]. In 2020, this combination was shown to be promising in treating H3K27M-mutant DIPG, improving OS in this subgroup of patients too [32].

With regard to other systemic therapies, many agents have been recently investigated, with several clinical trials still ongoing (Table 1).

Panobinostat is a histone deacetylase (HDAC) inhibitor that has been shown to have a wide spectrum of anti-tumorigenic effects, including induction of cell cycle arrest, inhibition of angiogenesis, and apoptosis. Nowadays, this agent has undergone rapid clinical development and has been approved by the European Medicines Agency (EMA) and the U.S. Food and Drugs Administration (FDA) for the treatment of relapsed/refractory multiple myeloma [33,34,35,36]. With regard to DIPG, as pre-clinical animal studies showed significantly prolonged survival in mice treated with Panobinostat, this agent was rapidly moved into clinical trials.

A phase I trial is currently ongoing to test side effects and optimal dosage of Panobinostat in children with DIPG (NCT02717455), with encouraging although preliminary results reported in abstract form [37]. To further enhance the efficacy of this therapeutic agent, an ongoing study (NCT04804709) is also investigating the possibility of opening up the blood–brain barrier through focused ultrasound using microbubbles and neuro-navigator-controlled sonication to allow a greater concentration of drugs to reach the tumor. Combination therapies such as Panobinostat administered with re-irradiation are under investigation, with a study published in 2017 reporting this combination being well tolerated at a relatively higher dose than that used in adult studies [38].

Another recently investigated therapeutic agent is the ONC201 molecule, which works by antagonizing the D2 dopamine receptor (DRD2) and deactivating Akt and ERK kinases. This phenomenon causes an increased expression of TRAIL in the nucleus, driven by Foxo3a, inducing a stress response and finally the death of the cancer cell [39].

Since the approval of ONC201, patients who have received this therapeutic agent following radiation therapy have shown a median OS of approximately 21.7 months, with a small percentage of them showing complete regression as well [40,41]. Considering the promising potential in the clinical setting, this agent is presently being investigated by a phase III study and it is currently being considered by the FDA for approval, which would make it the first FDA-approved therapeutic agent specific for patients with H3K27M-mutant gliomas [42]. Furthermore, the possibility of combining ONC201 with other drugs such as Panobinostat or Paxalisib is currently under investigation in a clinical trial (NCT05009992).

ONC206 is an example of a newer compound with a similar mechanism of action. This agent is considered to be a more potent blocker of dopamine receptors compared to ONC201, and it is also currently under investigation.

Following the success of immunotherapeutic agents for the treatment of hematologic malignancies, agents such as chimeric antigen receptor (CAR) T cells are currently under investigation for DIPG [42]. Indeed, following the recent observation that patient-derived H3K27M-mutant cell cultures and human tumor tissue had high expression of disialoganglioside GD2, this target has been tested as a potential antigen for CAR T-cell therapy in DIPG, showing promising results [43].

Gangliosides are glycosphingolipids, a class of lipidic structures containing galactose. These structures are normally found on the cellular surface of the mammalian nervous system, although widely expressed across different kinds of healthy tissues. Even if most gangliosides are considered to be ineffective as therapeutic targets, the research identified a unique target, namely GD2, which has been found to be over-expressed in several solid tumors (e.g., osteosarcoma, neuroblastoma, and gliomas) [44].

In this regard, Dinutuximab, which belongs to the class of anti-GD2 antibodies, was approved in 2015 by the FDA for the treatment of neuroblastoma, leading to an increased interest in anti-GD2 therapies [45]. Nowadays, clinical trials are underway to evaluate the efficacy and safety of CAR T-cell therapy. In this context, a phase 1 study (NCT04196413) has recently been conducted, with the aim of identifying the optimal dose, delivery method, and efficacy of GD2 CAR T cells, administered intravenously (IV) and intracerebroventricularly (ICV), in patients with H3 K27M-mutant DIPG or spinal cord diffuse midline glioma. Early results of this experience from 4 patients were published in 2022, while final results from 13 patients were published in 2024 [46,47,48]. Comprehensively, the authors highlighted the efficacy of this treatment strategy in obtaining tumor size decrement and neurological function improvements. In detail, radiologically proven tumor reduction has been identified in four patients (volumetric decrease of 52%, 54%, 91%, and 100%, respectively), with one of them obtaining a complete response for over 30 months from the beginning of the treatment. These radiological findings were found to be related to clinical benefits, with eight patients showing neurological improvements, according to a protocol-directed clinical improvement score. With regard to the safety profile, 1e6 GD2-CAR T/kg was identified as the maximally tolerated dose for IV administration while no dose-limiting toxicities were identified for ICV infusion (nine patients). Furthermore, although tumor inflammation-associated neurotoxicity was exhibited by all patients, this event was manageable in all cases with intensive monitoring and appropriate treatment [46,47,48].

Overall, GD2-CAR T cells offer several potential advantages over Dinutuximab for H3K27M gliomas, including improved tumor penetration, autonomous cytotoxicity, adaptability to tumor heterogeneity, and long-term persistence. However, careful consideration of safety and glioma-specific challenges will be crucial to unlocking their full potential. Specifically, although the management of adverse events related to the administration of GD2-CAR T-cell therapy remains a primary concern, this agent does represent an innovative approach with the potential to improve current outcomes for H3K27-mutant DIPGs and diffuse midline gliomas.

## 5. Radiotherapy for Local or Disseminated Disease

The mainstay for treating newly diagnosed DIPG is external beam radiotherapy, delivered in daily fractions of 1.8–2 Gy to a total dose of 54–60 Gy, over 6 weeks [49,50].

The most common delivery method is photon beam radiotherapy; however, protons represent a valid alternative, due to their advantage of releasing very high-dose gradients close to organs at risk, avoiding them, and respecting their dose constraints [51].

With regard to radiotherapy techniques, Intensity-Modulated Radiation Therapy (IMRT) is generally considered more advantageous compared to 3D Conformal Radiation Therapy (3D-CRT) [52,53]. IMRT’s precision is particularly important for DIPG, given its location in the brainstem, where sparing healthy tissue is crucial to reduce side effects and improve QoL. However, the choice between 3D-CRT and IMRT can depend on various factors, including the specific case, available technology, and the expertise of the treatment team.

According to SIOP-E recommendations, at disease progression, re-irradiation can be considered when eligibility criteria are fulfilled, and radio necrosis is excluded after upfront radiotherapy [54]. In this setting, different schemes have been investigated (24 Gy in 12 fractions, 26.4 Gy in 12 fractions, and 30.8 Gy in 14 fractions) with 24 Gy in 12 fractions being the preferred schedule [55].

Regarding the possibility of delivering higher radiotherapy doses, a large series of 20 patients, treated in a single institution, were recruited to evaluate a response-based dose escalation approach, showing better outcomes in terms of survival, with acceptable toxicity, for the second course of radiotherapy with a median total dose of 41.4 Gy, ranging from 33.8 to 43.2 Gy [56].

With regard to ongoing trials, a fractionation schedule of 20 Gy in 10 fractions at first re-irradiation in patients with diffuse midline gliomas is currently being investigated by the REMIT (RE-irradiation of diffuse MIdline glioma paTients) study (NCT06093165) with a planned extended follow-up on toxicity, performance status, and QoL [57].

Additionally, the possibility of delivering split-course radiation therapy is under investigation in the SPORT-DMG study (NCT05077735), which aims to change the way radiation is administered, delivering radiation over 2 weeks instead of the traditional 6 weeks of treatment [58].

Besides local recurrence, distant progression with leptomeningeal dissemination has been reported in up to 30% of cases. However, this percentage is believed to be underestimated due to low rates of imaging or autopsies performed at the end of life. In this regard, a study examining autopsy material from 16 DIPG patients observed subventricular dissemination in 63% of the patients, supporting the hypothesis that the real overall incidence of at-distance dissemination could be higher than reported [59,60].

In this setting, radiotherapy with craniospinal irradiation could be potentially performed to control both disease compartments. In the study reported by Somarriba et al., CSI was performed with a total dose of 21.6 Gy and was associated with good tolerance and clinical improvement. In the study by Massimino et al., CSI was performed with a total dose of 36 Gy [59,61].

With regard to our experience, we recently conducted a retrospective study on three children treated at our institution with systemic therapy (nimotuzumab and vinorelbine) and two or three courses of local irradiation [3].

The first radiotherapy course consisted of CFRT with a total dose of 54 Gy delivered in 27 fractions. The second course was delivered with a total dose of 19.8 Gy in 11 fractions. The third course was delivered with a total dose of 12 Gy in six fractions, with a cumulative BED10 of 102.6 Gy, and a cumulative BED3 of 141.7 Gy. In all children, neurological improvements were noticed after each radiotherapy course and were also associated with a radiological response. In this experience, the median OS was about 19.6, reaching 24 months in a child who, having the longest time to first and second progression, had the chance to be treated with three courses of radiotherapy [3].

Although educational, the significance of the above-mentioned experience is limited by the absence of molecular pathology. In fact, since the discovery of the hallmark H3K27M alteration as the major driver of DIPG, incorporating stereotactic biopsy into the diagnostic protocols for children with DIPG is considered crucial. This allows for the investigation of possible correlations between genetic alterations, treatment response, patterns of recurrence, and their impact on survival.

**Figure 1:**
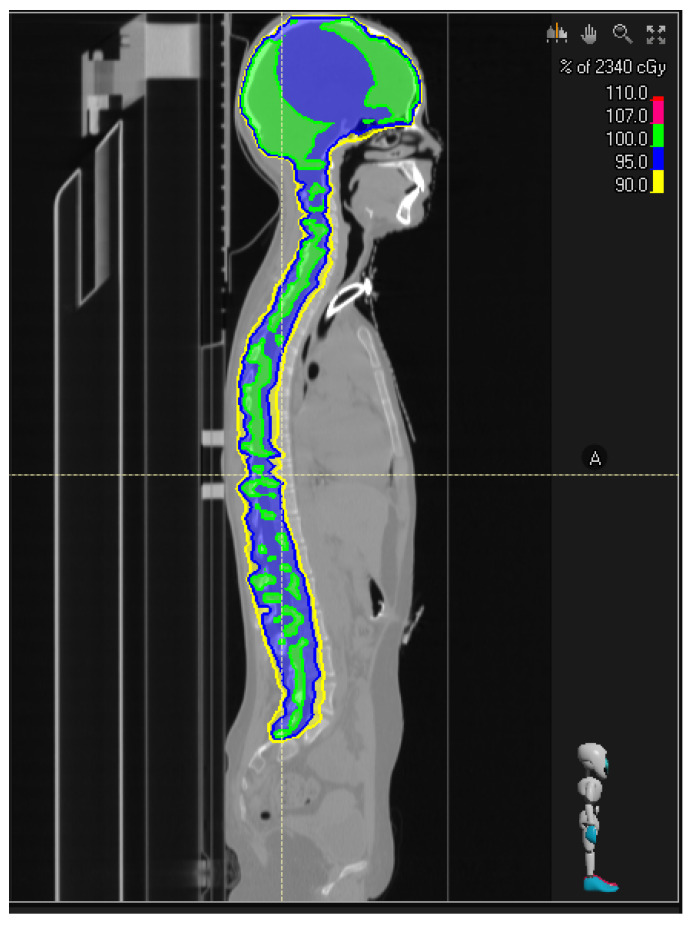
Craniospinal irradiation for improving control of leptomeningeal department.

Beyond the time to first recurrence, specific patterns of tumor recurrence, such as leptomeningeal dissemination, can influence treatment strategies. For example, tailoring radiotherapy based on tumor aggressiveness in terms of dose and volume is possible. Given the higher malignancy associated with the H3K27M mutation profile, a slightly increased dose could hypothetically be used for the second course of local irradiation, while irradiation of the entire craniospinal axis could help control both local and leptomeningeal compartments (Figure 1).

## 6. Conclusions

The introduction of stereotactic biopsy in the diagnostic pathway of Diffuse Intrinsic Pontine Glioma (DIPG) has been pivotal in enhancing our understanding of this aggressive malignancy and in fostering the development of innovative treatment strategies. Among systemic therapies, combinations such as nimotuzumab and vinorelbine, Panobinostat, ONC201, and CAR T-cell therapies are being explored, with many of these agents currently under investigation. Radiotherapy remains the cornerstone for both primary and recurrent DIPG, offering the potential to tailor treatment based on tumor characteristics, including doses and volumes, thereby optimizing therapeutic outcomes.

## Figures and Tables

**Table 1:** List of systemic therapies under investigation for DIPGs and diffuse midline gliomas.

	Approval	Trials	Status	Age
Nimotuzumab	Not yet approved by FDA or EMA	NCT04532229	Recruiting	3–15 years old
		NCT03620032	Active, not recruiting	2–21 years old
		NCT00561691	Completed	3–20 years old
		NCT00600054	Completed	3–18 years old
Panobinostat	EMA- and FDA-approved for the treatment of multiple myeloma	NCT04341311	Terminated	<22 years old
		NCT02717455	Completed	2–22 years old
		NCT03632317	Withdrawn	2–30 years old
		NCT03566199	Completed	2–21 years old
		NCT04804709	Active, not recruiting	4–21 years old
ONC201	Not yet approved by FDA or EMA It has EMA orphan designation for the treatment of glioma in E.U. and it is FDA Fast Track Designation-granted for H3K27M-mutant glioma and glioblastoma	NCT05476939	Recruiting	≥6 months old
		NCT05580562	Recruiting	Not reported
		NCT02525692	Active, not recruiting	≥16 years old
		NCT05009992	Recruiting	2–39 years old
ONC206	Not yet approved by FDA or EMA	NCT04732065	Recruiting	2–21 years old
		NCT04541082	Recruiting	≥18 years old
B7H3 CAR T cell	Not yet approved by FDA or EMA	NCT06221553	Recruiting	1–18 years old
		NCT04185038	Recruiting	1–26 years old
		NCT05768880	Recruiting	1–26 years old
		NCT05835687	Recruiting	≤21 years old
GD2 CAR T cell	Dinotuximab is the only GD2 CAR T-cell therapy that is FDA- and EMA-approved for the treatment of high-risk neuroblastoma in children.	NCT04196413	Recruiting	2–50 years old
		NCT04099797	Recruiting	1–22 years old
		NCT05544526	Recruiting	2–16 years old
		NCT05298995	Recruiting	6 months–30 years old
